# Cerebral Hemodynamic Changes in Older Adults With Cognitive Complaints According to the Presence of Peripheral Artery Disease

**DOI:** 10.1002/agm2.70121

**Published:** 2026-10-10

**Authors:** SangMi Noh, Hae‐Eun Shin, YongSoo Shim

**Affiliations:** ^1^ Department of Neurology, St. Vincent's Hospital, College of Medicine The Catholic University of Korea Seoul Republic of Korea; ^2^ Department of Neurology, Bucheon St. Mary's Hospital, College of Medicine The Catholic University of Korea Seoul Republic of Korea

**Keywords:** cerebral hemodynamics, peripheral artery disease, transcranial doppler

## Abstract

**Objective:**

As a systemic expression of atherosclerosis, peripheral artery disease (PAD) has the potential to alter cerebral hemodynamics in elderly individuals presenting with cognitive concerns. However, how PAD status shapes the longitudinal trajectory of cerebral hemodynamics has not been well characterized.

**Methods:**

We conducted a single‐center longitudinal observational study enrolling 132 participants (21 controls, 39 mild cognitive impairment, 72 mild Alzheimer's disease). All participants underwent transcranial Doppler (TCD) ultrasonography, ankle–brachial index (ABI) measurement, brain MRI, and cognitive assessments at baseline and follow‐up (~13.45 months). PAD was defined as ABI < 0.90. Bilateral mean flow velocity (MFV), pulsatility index (PI), and resistance index (RI) of the middle cerebral artery (MCA) were tracked longitudinally across both time points.

**Results:**

PAD was identified in 13 participants (9.8%). Baseline TCD parameters, vascular risk factors, white matter lesions on brain MRI, and cognitive measures were comparable between groups. At follow‐up, the PAD group showed significantly higher MCA MFV after age adjustment (left *p* < 0.001; right *p* = 0.005). MFV increased in the PAD group (left +16.45 cm/s) but decreased in the non‐PAD group (left −3.37 cm/s), with a significant between‐group difference. PI and RI did not change in either group. Cognitive and functional outcomes were similar across groups throughout the study.

**Conclusion:**

These findings identify PAD as a condition associated with progressive MCA MFV elevation in the context of preserved cognition, implying that subclinical cerebrovascular changes are detectable by TCD before overt clinical worsening. Future research involving larger cohorts and extended follow‐up is required to determine whether such hemodynamic changes forecast eventual cognitive deterioration.

## Graphical abstract

Among 132 older adults, 13 had peripheral artery disease (PAD), identified using the ankle–brachial index. Over approximately 13 months, PAD was associated with increasing middle cerebral artery mean flow velocity, with a significant difference in left‐sided velocity change between groups. Pulsatility and resistance indices remained unchanged, while cognitive and functional measures showed no significant differences between groups. These findings suggest that systemic vascular pathology may alter cerebral hemodynamics without concurrent measurable cognitive decline, warranting further investigation in larger longitudinal studies.

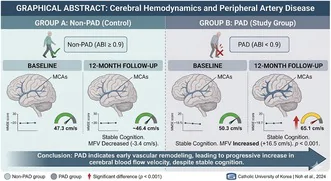

## 1 Introduction

Vascular pathology is an established risk factor for cognitive decline in older individuals. In particular, cerebral small vessel disease, typically visualized as white matter hyperintensities (WMHs) on brain magnetic resonance imaging (MRI), has been associated with a heightened risk of cognitive impairment and dementia [1, 2]. Transcranial Doppler (TCD) ultrasonography enables noninvasive assessment of cerebral hemodynamics. TCD‐derived measurements of blood flow velocity in the intracranial arteries reliably reflect cerebral blood flow status [3, 4], and have also proven useful for assessing cerebrovascular microvascular integrity in aging populations [5, 6].

Beyond cerebral small vessel disease, other forms of systemic vascular dysfunction may also affect cerebral hemodynamics and cognitive function [7, 8]. Peripheral artery disease (PAD), a type of systemic atherosclerosis, affects a significant portion of the elderly population [9, 10], and is one such condition. PAD indicates widespread endothelial dysfunction and systemic vascular burden [11], suggesting that individuals with PAD might experience changes in cerebral blood flow regulation [12]. The ankle–brachial index (ABI), used as a noninvasive screening tool for PAD, shows that a reduced ABI (< 0.9) confers heightened cardiovascular and cerebrovascular risk as well as increased susceptibility to cognitive impairment [7, 8, 13, 14, 15].

While the adverse effects of PAD on physical function and cardiovascular health are well established, the nature of its relationship with cerebral hemodynamics and cognitive performance is less certain. Prior studies investigating associations between PAD and cognitive impairment have produced conflicting findings, and direct assessments of cerebral hemodynamic parameters in relation to PAD remain limited [12]. Consequently, the pathways through which peripheral vascular disease influences cerebral circulation warrant further elucidation.

To address these gaps, we hypothesized that individuals with cognitive complaints and PAD may exhibit altered cerebral hemodynamic responses compared to those without PAD. Accordingly, this study aimed to evaluate cerebral hemodynamics assessed by TCD in older adults with memory complaints according to PAD status defined by ABI, and to examine their associations with clinical and cognitive functions.

## 2 Materials and Methods

### 2.1 Participants

This single‐center observational study was approved by the Institutional Review Board of The Catholic University of Korea, Bucheon St. Mary's Hospital, and all subjects provided written informed consent. We consecutively enrolled patients attending the hospital's Neurology outpatient clinic. The study comprised 132 participants: 21 controls, 39 patients with mild cognitive impairment (MCI), and 72 patients with mild Alzheimer's disease (AD). Healthy adults seeking evaluation for possible cerebrovascular disease due to concerns about stroke or vascular risk factors were enrolled as controls. Controls reported no cognitive complaints, scored within the normal range on the Korean version of the Mini‐Mental State Examination (K‐MMSE) [16] (within 1.0 standard deviation of age‐ and education‐matched norms), and had a Clinical Dementia Rating (CDR) [17] score of zero. MCI was diagnosed according to established clinical criteria [18]. Probable AD was diagnosed using NINCDS‐ADRDA criteria [19].

All participants completed physical and neurological examinations, laboratory tests (complete blood count, blood chemistry, vitamin B12/folate, syphilis serology), thyroid function assessment, K‐MMSE‐based cognitive screening, brain MRI and MR angiography (MRA), and ABI measurement. A vascular risk factor history including hypertension, diabetes mellitus, hyperlipidemia, ischemic heart disease, stroke, and smoking was obtained for each participant. Exclusion criteria included large territorial infarcts on MRI, age below 55 years, conditions other than MCI or AD capable of causing cognitive impairment, and major psychiatric illness. Time‐of‐flight MRA was reviewed to identify intracranial large‐artery stenosis. Participants with significant stenosis (≥ 50% luminal narrowing) in major cerebral arteries were excluded to prevent focal structural lesions from confounding TCD‐derived flow velocity measurements.

### 2.2 MRI Assessment

All participants underwent 3.0T brain MRI (Intera; Philips Medical Systems, Best, The Netherlands) encompassing susceptibility‐weighted imaging, fluid‐attenuated inversion recovery (FLAIR), and T1‐ and T2‐weighted sequences acquired with the spin‐echo technique. Slice thickness was 5 mm with no interslice gap. MRA was performed using a three‐dimensional time‐of‐flight technique. Periventricular WMHs (PVHs) and deep WMHs (DWMHs) were separately graded according to the Clinical Research for Dementia of South Korea (CREDOS) criteria [20]. DWMH severity was classified by largest lesion diameter: D1 (< 10 mm), D2 (≥ 10 and < 25 mm), and D3 (≥ 25 mm). PVHs were graded P1 (cap and band < 5 mm), P2 (cap or band ≥ 5 and < 10 mm), or P3 (cap or band ≥ 10 mm). Total WMH severity was categorized as none (Grade 0), minimal (Grade 1), moderate (Grade 2), or severe (Grade 3) [20]. On susceptibility‐weighted imaging, CMBs were defined as focal signal‐loss areas smaller than 10 mm. Mimics including calcifications, venous structures, blood vessels, and cavernous malformations were excluded from CMB counts. Signal voids from sulcal vessels or symmetric basal ganglia, choroid plexus, or pineal calcifications were not counted [21, 22]. Lacunar infarcts were identified as T2‐hyperintense, T1‐hypointense lesions (3–15 mm) with a perilesional FLAIR halo [23]. The total count of lacunae and CMBs was recorded. Medial temporal atrophy was scored on a 5‐point scale (0–4) on T1‐weighted coronal images based on the width of the choroidal fissure and temporal horn and height of the hippocampal formation [24].

### 2.3 Assessment of PAD

ABI was determined using an oscillometric device (form PWV/ABI, COLIN, Tokyo, Japan). Mechano‐cardiograms were simultaneously recorded using blood pressure cuffs equipped with topometric sensors attached to the upper arm and ankle. ABI was calculated as the ratio of ankle to brachial systolic blood pressure, measured bilaterally with participants supine. The high ankle pressure method was used. The higher of the dorsalis pedis and posterior tibial systolic pressures for each leg was divided by the higher brachial systolic pressure [25]. PAD was defined as ABI < 0.90 in either leg, per established diagnostic guidelines [26].

### 2.4 TCD Measurements

All participants were evaluated with TCD ultrasonography. The examination used a 2‐MHz Doppler probe (Viasys Healthcare, Model Sonara) applied through the temporal bone window by a sonographer blinded to the clinical diagnosis. Participants were positioned supine with the probe fixed at the temporal window. Bilateral proximal MCA segments at depths of 55–65 mm were insonated, and MFV was recorded. MCA flow velocity changes reliably correlate with changes in cerebral blood flow [27]. The pulsatility index (PI) and resistance index (RI) were also derived. PI was computed as (peak systolic velocity − end‐diastolic velocity)/MFV; it reflects distal flow resistance and vascular wall rigidity, analogous to pulse pressure. RI, a measure of peripheral flow resistance, was calculated as (peak systolic velocity − end‐diastolic velocity)/peak systolic velocity.

### 2.5 Follow‐Up

Approximately 12 months later (13.45 ± 3.62 months), each participant underwent follow‐up clinical evaluation including the same neuropsychological tests (K‐MMSE, CDR, and CDR‐sum of boxes (CDR‐SB)) and TCD ultrasonography to assess hemodynamic markers (MFV, PI, and RI). All measurements were conducted by an experienced sonographer blinded to the ABI results and cognitive status. Follow‐up evaluation did not include repeat brain MRI; consequently, new ischemic lesions or other structural cerebrovascular changes occurring during the follow‐up interval could not be assessed.

### 2.6 Statistical Analysis

Between‐group comparisons of demographic and clinical characteristics (age, education, sex, apolipoprotein E [APOE] genotype, vascular risk factors, Hachinski ischemic score, BMI), baseline MRI findings (WMHs), cognitive measures (MMSE and CDR), and TCD parameters (MFV, PI, and RI) at both time points were conducted between PAD and non‐PAD participants.

Continuous data are reported as mean ± standard deviation, and categorical data as counts and percentages. Data normality was evaluated using the Kolmogorov–Smirnov test. Unadjusted between‐group comparisons used the Mann–Whitney *U* test or independent *t*‐test for continuous variables and the chi‐square or Fisher's exact test for categorical variables, as appropriate. Given the age discrepancy between the two groups, analysis of covariance (ANCOVA) was applied to derive age‐adjusted *p* values for continuous comparisons, and logistic regression was used for categorical variables. Age‐adjusted analyses are treated as primary results and are reported in the tables.

Longitudinal changes in cognitive and hemodynamic parameters were evaluated by comparing follow‐up values and changes from baseline (Δ) between PAD and non‐PAD groups using age‐adjusted ANCOVA. Within‐group changes from baseline to follow‐up were assessed using paired *t*‐tests and are summarized in the text. Medication information (type and number of cardiovascular medications) was recorded for all participants at baseline and follow‐up but was not formally included as a covariate in the primary statistical models due to the small size of the PAD group.

All statistical tests were two‐tailed, and statistical significance was set at *p* < 0.050. Statistical analyses were performed using the Statistical Package for the Social Sciences version 30.0 (IBM Corp., Armonk, NY, USA).

## 3 Results

### 3.1 Baseline Characteristics and Assessments

Of the 132 participants, 13 (9.8%) were diagnosed with PAD based on an ABI < 0.90. The PAD group tended to be older than the non‐PAD group (mean age 76.85 ± 5.29 vs. 72.58 ± 7.40 years, *p* = 0.054). There were no significant differences between the two groups in sex distribution, years of education, BMI, or vascular risk factors including hypertension, diabetes mellitus, hyperlipidemia, ischemic heart disease, smoking, and previous stroke history.

At baseline, global cognitive measures did not differ between groups. MMSE, CDR, and CDR‐SB scores were comparable between the PAD and non‐PAD groups after age adjustment (Table 1). MRI assessments showed no significant differences in WMH burden. Similarly, baseline TCD parameters—including MFV, PI, and RI in both MCAs—did not differ significantly between groups. No participant included in the final analysis had significant intracranial large‐artery stenosis on MRA. Baseline TCD was performed in all participants (*n* = 119 non‐PAD; *n* = 13 PAD). At follow‐up, TCD data were available for 114 of 119 non‐PAD and 12 of 13 PAD participants; 5 non‐PAD and 1 PAD participant were lost to follow‐up.

**TABLE 1:** Demographic and clinical characteristics of the study participants.

Variable	Non‐PAD (*n* = 119)	PAD (*n* = 13)	*p*
Diagnostic group (control:MCI:AD)	20:36:63	20:36:63	0.563
Age, years	72.58 ± 7.40	76.85 ± 5.29	0.054
Education (years)	6.97 ± 4.84	6.00 ± 5.87	0.832
Sex, male (%)	35 (29.4)	6 (46.2)	0.223
Body mass index (kg/m^2^)	23.01 ± 2.84	22.48 ± 2.25	0.487
Hachinski Ischemic Scale	1.99 ± 1.41	3.23 ± 2.71	0.021
APOE ε4 carrier^a^	40/107 (37.4)	3/12 (25.0)	0.872
Vascular risk factors			
Hypertension	85 (71.4)	8 (61.5)	0.525
Diabetes mellitus	35 (29.4)	4 (30.8)	0.572
Hyperlipidemia	62 (52.1)	7 (53.8)	0.570
Ischemic heart disease	10 (8.4)	4 (30.8)	0.033
Previous stroke	13 (10.9)	2 (15.4)	0.645
Smoking history	10 (8.4)	3 (23.1)	0.119
Cognitive measures			
MMSE score	20.40 ± 6.65	18.46 ± 5.38	0.776
CDR score	0.790 ± 0.690	1.12 ± 0.870	0.209
CDR‐SB score	4.08 ± 3.99	6.19 ± 5.58	0.369
GDS score	3.52 ± 1.07	4.45 ± 1.04	0.016
MRI parameters			
Deep WMH grade	0.860 ± 0.830	1.08 ± 0.760	0.625
Periventricular WMH grade	1.63 ± 1.05	2.08 ± 0.760	0.469
Baseline TCD parameters			
MFV, left MCA (cm/s)	47.29 ± 12.93	50.31 ± 10.74	0.257
MFV, right MCA (cm/s)	47.29 ± 13.52	52.58 ± 15.75	0.119

*Note:* Values are presented as mean ± standard deviation or *n* (%) unless otherwise indicated. All *p* values shown are age‐adjusted. Age‐adjusted *p* values were obtained using analysis of covariance (ANCOVA) for continuous variables and logistic regression for categorical variables.

Abbreviations: CDR, Clinical Dementia Rating; CDR‐SB, CDR‐sum of boxes; GDS, Geriatric Depression Scale; MCA, middle cerebral artery; MFV, mean flow velocity; MMSE, Mini‐Mental State Examination; PAD, peripheral artery disease; WMH, white matter hyperintensity.

^a^ APOE genotyping was available in 107 participants without PAD and 12 participants with PAD.

### 3.2 Longitudinal Outcomes

The mean follow‐up duration was similar between the PAD and non‐PAD groups (13.57 ± 3.74 vs. 12.35 ± 1.95 months). At follow‐up, no significant between‐group differences were observed in MMSE, CDR, or CDR‐SB scores after age adjustment, indicating minimal longitudinal change in global cognitive function in either group (Table 2). Individual longitudinal trajectories of MMSE, CDR, and CDR‐SB scores according to PAD status are presented in Figure S1.

**TABLE 2:** Baseline, follow‐up, and longitudinal changes in TCD and cognitive parameters according to PAD status.

Variable	Non‐PAD (*n* = 119)	PAD (*n* = 13)	*p*
Baseline			
MMSE score	20.40 ± 6.65	18.46 ± 5.38	0.776
CDR score	0.790 ± 0.690	1.12 ± 0.870	0.209
CDR‐SB score	4.08 ± 3.99	6.19 ± 5.58	0.191
MFV, left MCA (cm/s)	47.29 ± 12.93	50.31 ± 10.74	0.257
MFV, right MCA (cm/s)	47.29 ± 13.52	52.58 ± 15.75	0.119
Follow‐up			
Duration (months)	13.57 ± 3.74	12.35 ± 1.95	—
MMSE score	20.32 ± 6.88	18.67 ± 6.04	0.887
CDR score	0.870 ± 0.780	1.04 ± 0.620	0.764
CDR‐SB score	4.73 ± 4.64	5.79 ± 4.55	0.852
MFV, left MCA (cm/s)	46.41 ± 13.58	65.11 ± 27.43	< 0.001
MFV, right MCA (cm/s)	49.05 ± 14.75	62.44 ± 28.40	0.005
Change from baseline (Δ)			
ΔMMSE score	−0.120 ± 3.74	−0.500 ± 3.92	0.678
ΔCDR score	0.050 ± 0.430	0.080 ± 0.560	0.937
ΔCDR‐SB score	0.4009 ± 2.29	0.580 ± 3.78	0.997
ΔMFV, left MCA (cm/s)	−3.37 ± 15.68	16.45 ± 19.41	< 0.001
ΔMFV, right MCA (cm/s)	−0.270 ± 18.45	10.91 ± 14.47	0.058

*Note:* Values are presented as mean ± standard deviation. All *p* values shown are age‐adjusted. Age‐adjusted *p* values were obtained using analysis of covariance (ANCOVA). Δ values represent follow‐up minus baseline. Follow‐up TCD data were available for 114 of 119 non‐PAD and 12 of 13 PAD participants (5 non‐PAD and 1 PAD participant were lost to follow‐up). Longitudinal MFV analyses for the left MCA included participants with paired measurements (*n* = 106 for non‐PAD, *n* = 11 for PAD); right MCA analyses included *n* = 110 non‐PAD and *n* = 12 PAD participants with paired data.

Abbreviations: CDR, Clinical Dementia Rating; CDR‐SB, CDR‐sum of boxes; MCA, middle cerebral artery; MFV, mean flow velocity; MMSE, Mini‐Mental State Examination; PAD, peripheral artery disease.

In contrast to the stable cognitive measures, marked between‐group differences emerged in cerebral hemodynamic parameters over time. At follow‐up, participants with PAD exhibited significantly higher absolute MFV values in both the left MCA (65.11 ± 27.43 vs. 46.41 ± 13.58 cm/s, age‐adjusted *p* < 0.001) and the right MCA (62.44 ± 28.40 vs. 49.05 ± 14.75 cm/s, age‐adjusted *p* = 0.005) compared with the non‐PAD group (Table 2).

Analysis of longitudinal changes further demonstrated divergent MFV trajectories between groups. Over the follow‐up period, MFV increased substantially in the PAD group (left MCA: +16.45 ± 19.41 cm/s; right MCA: +10.91 ± 14.47 cm/s), whereas MFV decreased or remained stable in the non‐PAD group (left MCA: −3.37 ± 15.68 cm/s; right MCA: −0.27 ± 18.45 cm/s). The between‐group difference in MFV change was statistically significant for the left MCA (age‐adjusted *p* < 0.001) and showed a trend toward significance for the right MCA (age‐adjusted *p* = 0.058) (Figure 1). Individual longitudinal trajectories of bilateral MCA MFV according to PAD status are illustrated in Figure S2. No significant longitudinal changes or between‐group differences were observed in PI or RI in either MCA during the follow‐up period (data not shown).

**FIGURE 1:**
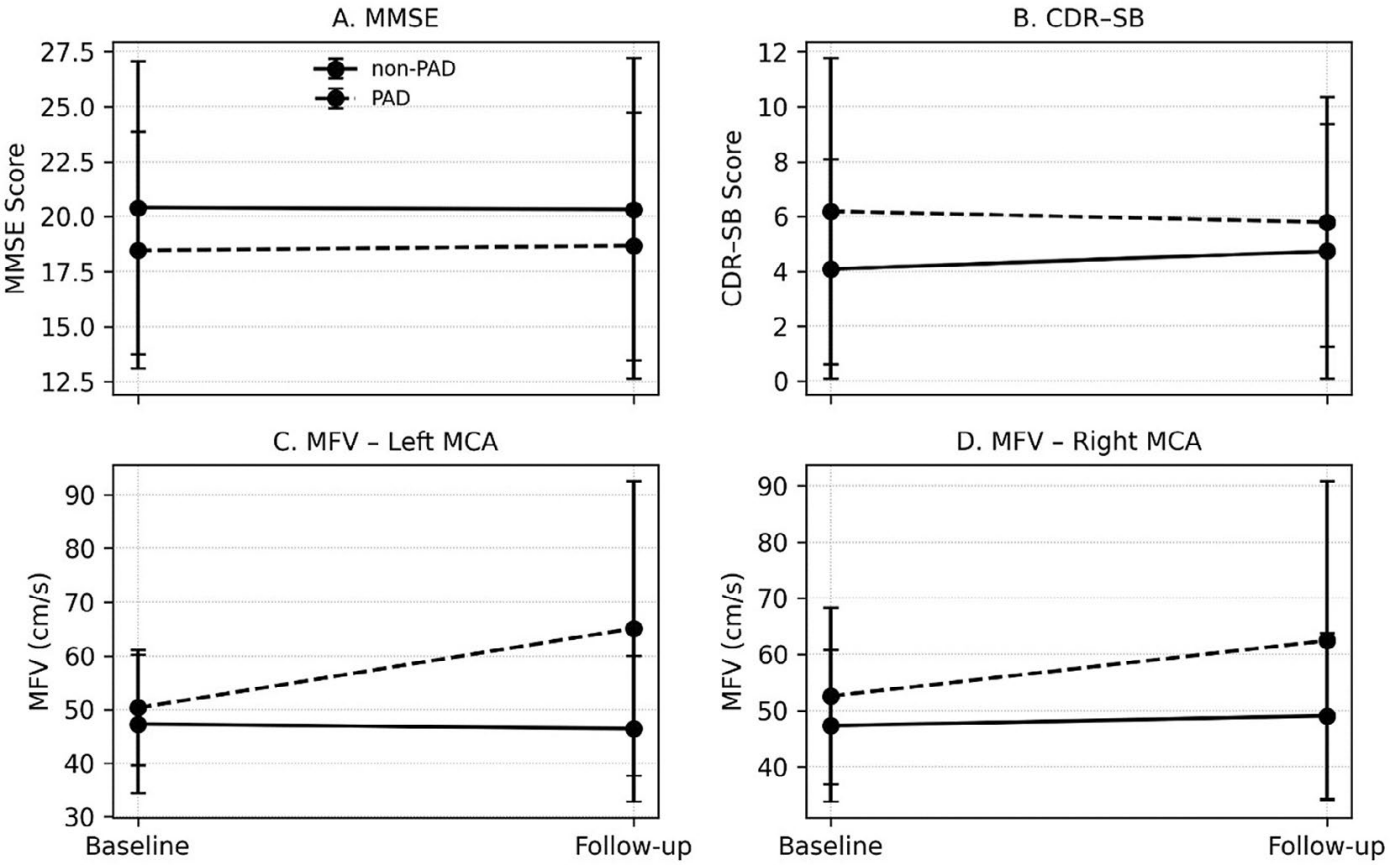
Longitudinal changes in cognitive and cerebral hemodynamic measures according to the presence of peripheral artery disease (PAD). (A) Mini‐Mental State Examination (MMSE) and (B) Clinical Dementia Rating–Sum of Boxes (CDR‐SB) scores remained relatively stable over time in both PAD and non‐PAD groups. (C) Mean flow velocity (MFV) in the left middle cerebral artery (MCA) increased in the PAD group but decreased in the non‐PAD group (between‐group difference in change, *p* < 0.001). (D) A similar pattern was observed in the right MCA (*p* = 0.036). Data are presented as mean ± standard deviation. Solid lines indicate the non‐PAD group, and dashed lines indicate the PAD group.

Derived peak systolic velocity (PSV) and end‐diastolic velocity (EDV), calculated from recorded MFV, PI, and RI values, are provided in Table S1 and showed directionally consistent increases in the PAD group (left MCA: PSV from 92.2 ± 24.5 to 120.2 ± 53.7 cm/s; right MCA: PSV from 100.8 ± 32.2 to 121.0 ± 61.9 cm/s). Individual MFV values for all PAD group participants are provided in Table S2, confirming that the overall increase was consistent across the majority of participants and was not driven by a single extreme case.

## 4 Discussion

This study examined how PAD, defined by ABI, relates to longitudinal changes in cerebral hemodynamics in elderly adults with cognitive complaints. Despite comparable baseline MCA MFVs across groups, PAD participants showed a significant progressive MFV increase during follow‐up while non‐PAD participants remained hemodynamically stable, yielding a significant between‐group difference in the magnitude of MFV change. Critically, these divergent hemodynamic courses occurred in the absence of corresponding changes in global cognition or daily function, as gauged by MMSE and CDR.

The longitudinal increase in MFV among participants with PAD requires careful interpretation. In primary neurodegenerative conditions such as AD, cerebral blood flow velocity is typically reduced, reflecting neuronal loss and decreased metabolic demand [28, 29]. In contrast, the observed increase in MFV in the PAD group suggests a distinct vascular mechanism rather than progressive neurodegeneration. This pattern is unlikely to represent improved cerebral perfusion. Instead, it more plausibly reflects systemic atherosclerosis‐related vascular remodeling, including arterial stiffening, reduced compliance, and compensatory flow acceleration within large cerebral arteries [30, 31, 32].

Because all participants with ≥ 50% intracranial large‐artery stenosis were excluded based on MRA findings, the observed MFV increase cannot be attributed to overt focal luminal narrowing and more likely reflects diffuse vascular remodeling or functional hemodynamic alterations. Rather, PAD‐associated systemic vascular pathology may impose early functional hemodynamic stress on cerebral arteries, resulting in increased flow velocity without substantial alterations in distal resistance. This interpretation is supported by the absence of significant longitudinal changes in PI and RI, indices more strongly influenced by downstream microvascular resistance and relatively insensitive to early or moderate vascular dysfunction [33, 34, 35, 36].

Notably, these hemodynamic changes were not accompanied by measurable cognitive decline during the follow‐up period. This dissociation supports a temporal model in which PAD‐related vascular pathology first manifests as altered cerebral hemodynamics, preceding overt cognitive deterioration. PAD, as a marker of systemic atherosclerosis and endothelial dysfunction, may impair cerebral autoregulation and expose the brain to chronic hemodynamic stress. Over time, reduced vascular reserve and impaired perfusion efficiency may increase neuronal vulnerability and interact with neurodegenerative processes. Prior studies linking cerebral blood flow abnormalities to structural brain changes and cerebrovascular lesions further support this vascular vulnerability framework [37, 38, 39, 40].

Clinically, these findings underscore the importance of systemic vascular assessment in older adults presenting with cognitive complaints. PAD, a readily measurable but often underrecognized condition, may signal broader cerebrovascular susceptibility. Combined assessment of ABI and TCD‐derived hemodynamic parameters may provide complementary information regarding cerebral vascular status. In this context, increasing MFV in individuals with PAD may represent a transitional and potentially modifiable stage before clinically apparent cognitive decline.

Several limitations merit consideration. First, the relatively small number of participants with PAD may have limited statistical power and generalizability to broader populations with varying vascular risk profiles. Larger, adequately powered longitudinal cohorts are needed to determine whether PAD‐related MFV changes independently predict subsequent cognitive decline. Second, medication information was recorded for all participants at both baseline and follow‐up. Some participants across both groups were receiving cardiovascular medications; however, no significant difference in medication use was observed between the PAD and non‐PAD groups at baseline, and medication use remained unchanged throughout the follow‐up period. Nevertheless, cardiovascular medications—including antiplatelet agents, antihypertensives, and statins—may independently influence cerebral hemodynamic parameters, and their potential confounding effect could not be fully excluded from the current analysis. Future studies with larger cohorts should systematically examine whether specific medication classes modify the relationship between PAD and longitudinal cerebral hemodynamic changes. Third, although age was adjusted for, residual confounding cannot be excluded; blood pressure variability, metabolic and inflammatory status, and cardiac function may all influence cerebral hemodynamics and warrant more comprehensive control in future studies. Fourth, TCD measures flow velocity rather than absolute cerebral blood flow and does not directly quantify regional perfusion. The absence of structural neuroimaging and disease‐specific biomarkers further limits mechanistic differentiation between vascular and neurodegenerative contributions. Integration of multimodal perfusion imaging—such as arterial spin labeling or perfusion MRI—together with advanced TCD‐derived indices and established AD biomarkers may clarify the structural and functional correlates of MFV alterations. Furthermore, follow‐up brain MRI was not included in the study protocol; we were therefore unable to confirm whether new cerebrovascular lesions—including silent cerebral infarctions—occurred during the follow‐up period and potentially contributed to the observed MFV increase in the PAD group. None of the PAD participants were documented to have experienced a symptomatic clinical stroke during follow‐up; however, subclinical infarctions cannot be excluded without repeat imaging. Repeat neuroimaging in future longitudinal studies would be valuable to disentangle hemodynamic changes due to progressive vascular pathology from those attributable to discrete new structural events. Finally, longer follow‐up is required to determine whether the observed hemodynamic patterns represent transient adaptations or progressive trajectories leading to overt dementia or vascular cognitive impairment.

## 5 Conclusion

In summary, PAD was associated with a distinct longitudinal increase in MCA flow velocity that occurred independently of measurable cognitive decline. This dissociation suggests that systemic vascular pathology may alter cerebral hemodynamics at an early stage, preceding clinically apparent cognitive deterioration and potentially representing a modifiable window of cerebrovascular vulnerability.

## Author Contributions

**SangMi Noh:** methodology, writing – review and editing, conceptualization. **Hae‐Eun Shin:** data curation, writing – review and editing. **YongSoo Shim:** visualization, software, formal analysis, writing – review and editing, supervision, conceptualization.

## Funding

This study was supported in part by the financial support of the Catholic Medical Center Research Foundation made in the program year of 2012 and College of Medicine, Catholic University of Korea.

## Ethics Statement

This study is a retrospective analysis of the patients' clinical and TCD data, which was approved by the Institutional Review Board of the Catholic University of Korea, Bucheon St. Mary's Hospital. Subjects provided verbal informed consent. This study protocol was reviewed and approved by the Institutional Review Board of the Catholic University of Korea, Bucheon St. Mary's Hospital (Approval number HC12RISI0030).

## Conflicts of Interest

The authors declare no conflicts of interest.

## Supporting information


**Figure S1:** Individual longitudinal trajectories of (A) MMSE and (B) CDR‐SB scores from baseline to follow‐up in participants without PAD (left) and with PAD (right). Thin lines represent individual participants, color‐coded by baseline cognitive group, and thick lines indicate group‐wise mean values ± standard error. No significant differences in longitudinal cognitive or functional trajectories were observed according to PAD status.

**Figure S2:** Individual longitudinal trajectories of mean flow velocity (MFV) in the middle cerebral artery from baseline to follow‐up in participants without PAD (left) and with PAD (right). Baseline MFV values were comparable between groups, whereas longitudinal MFV trajectories diverged, with a greater increase observed in the PAD group. No significant PAD × cognitive group interaction was observed.

**Table S1:** Derived peak systolic velocity (PSV) and end‐diastolic velocity (EDV) of middle cerebral arteries at baseline and follow‐up according to PAD status.
**Table S2:** Individual mean flow velocity (MFV) values at baseline and follow‐up for participants in the PAD group.

## Data Availability

The data that support the findings of this study are not publicly available due to containing information that could compromise the privacy of research participants but are available from the corresponding author upon reasonable request.
