# Supplementary material for: Cerebral Hemodynamic Changes in Older Adults With Cognitive Complaints According to the Presence of Peripheral Artery Disease
Source: Aging Med (Milton). 2026 Oct 10:10.1002/agm2.70121. Online ahead of print. doi: 10.1002/agm2.70121 (PMC13653594; doi:10.1002/agm2.70121)
Supplement: Supplementary file 1 [file AGM2-9999-0-s003.docx]

**Supplementary Tables**

*Cerebral Hemodynamic Changes in Older Adults with Cognitive Complaints According to the Presence of Peripheral Artery Disease*

**Supplementary Table S1.** Derived Peak Systolic Velocity (PSV) and End-Diastolic Velocity (EDV) of Middle Cerebral Arteries at Baseline and Follow-up According to PAD Status

| **Variable** | **Non-PAD (n = 119)** | **PAD (n = 13)** | **p value** |
| --- | --- | --- | --- |
| **Baseline** |  |  |  |
| L MCA PSV (cm/s) | 80.3 +/- 20.0 | 92.2 +/- 24.5 | 0.088 |
| L MCA EDV (cm/s) | 26.1 +/- 9.4 | 25.8 +/- 6.6 | 0.924 |
| R MCA PSV (cm/s) | 85.6 +/- 27.8 | 100.8 +/- 32.2 | 0.096 |
| R MCA EDV (cm/s) | 24.2 +/- 9.8 | 27.8 +/- 7.0 | 0.209 |
| **Follow-up** |  |  |  |
| L MCA PSV (cm/s) | 78.5 +/- 24.2 | 120.2 +/- 53.7 | < 0.001 |
| R MCA PSV (cm/s) | 84.0 +/- 26.6 | 121.0 +/- 61.9 | 0.005 |

*Values are presented as mean +/- standard deviation. PSV and EDV are derived from recorded MFV, PI, and RI values using the formulas: PSV = PI x MFV / RI; EDV = PSV x (1 - RI). P values are age-adjusted (ANCOVA). Age-adjusted p values < 0.05 shown in bold.
PAD = peripheral artery disease; MCA = middle cerebral artery; PSV = peak systolic velocity; EDV = end-diastolic velocity; MFV = mean flow velocity; PI = pulsatility index; RI = resistance index.*

**Supplementary Table S2.** Individual Mean Flow Velocity (MFV) Values at Baseline and Follow-up for Participants in the PAD Group

| **Patient** | **ABI (lower)** | **L MCA Baseline (cm/s)** | **L MCA Follow-up (cm/s)** | **R MCA Baseline (cm/s)** | **R MCA Follow-up (cm/s)** |
| --- | --- | --- | --- | --- | --- |
|  |  | *(Δ from baseline)* |  | *(Δ from baseline)* |  |
| 1 | 0.84 | 56.6 | 64.5 (+7.9) | 50.4 | 66.7 (+16.3) |
| 2 | 0.73 | 42.4 | 48.9 (+6.5) | 56.2 | 55.6 (-0.6) |
| 3 | 0.55 | 52.7 | N/A† | 55.1 | N/A† |
| 4 | 0.9 | 62.2 | 86 (+23.8) | 52 | 60.4 (+8.4) |
| 5 | 0.9 | 34.3 | 35 (+0.7) | 37.3 | 38.1 (+0.8) |
| 6 | 0.83 | 56.2 | 67.4 (+11.2) | 62.4 | 62.4 (+0.0) |
| 7 | 0.79 | 39.7 | 46.6 (+6.9) | 38.1 | 38.1 (+0.0) |
| 8 | 0.9 | N/A‡ | N/A‡ | 32 | 39.7 (+7.7) |
| 9 | 0.83 | 50 | 83.8 (+33.8) | 66.6 | 84.9 (+18.3) |
| 10 | 0.87 | 40.8 | 24.5 (-16.3) | 40.8 | 33.5 (-7.3) |
| 11 | 0.9 | 59.1 | 116 (+56.9) | 65.4 | 95.8 (+30.4) |
| 12 | 0.9 | 43.1 | 48.5 (+5.4) | 50.2 | 38.1 (-12.1) |
| 13 | 0.46 | 65.8 | 91.5 (+25.7) | 94.7 | 127 (+32.3) |
| **Mean +/- SD (all)** | **--** | **50.2 +/- 10.1** | **--** | **53.9 +/- 16.4** | **--** |
| **Mean +/- SD (paired)*** | **--** | **50.0 +/- 10.5** | **64.8 +/- 27.4** | **53.8 +/- 17.2** | **61.7 +/- 28.6** |

*ABI = lowest ankle-brachial index value (right or left). Values in parentheses indicate change from baseline. † Patient 3: Lost to follow-up; no TCD measurement obtained at follow-up visit. ‡ Patient 8: Inadequate temporal bone window precluded left MCA assessment at baseline and follow-up. * Mean +/- SD for paired data only (patients with both baseline and follow-up measurements available). PAD = peripheral artery disease; MCA = middle cerebral artery; MFV = mean flow velocity; ABI = ankle-brachial index. Patients are listed in order of enrollment and are anonymized.*
